# Inhibition of receptor tyrosine kinase signalling by small molecule agonist of T-cell protein tyrosine phosphatase

**DOI:** 10.1186/1471-2407-10-7

**Published:** 2010-01-07

**Authors:** Elina Mattila, Heidi Marttila, Niko Sahlberg, Pekka Kohonen, Siri Tähtinen, Pasi Halonen, Merja Perälä, Johanna Ivaska

**Affiliations:** 1 VTT Technical Research Centre of Finland, Medical Biotechnology, Itainen Pitkakatu 4B, FIN-20520 Turku, Finland; 2 Centre for Biotechnology, University of Turku, Tykistokatu 6, FIN-20520 Turku, Finland; 3 Department of Biochemistry and Food Chemistry, University of Turku, Vatselankatu 2, FIN-20014 Turku, Finland

## Abstract

**Background:**

T-cell protein tyrosine phosphatase (TCPTP/TC45) is a ubiquitously expressed intra-cellular non-receptor protein tyrosine phosphatase involved in the negative regulation of several cancer relevant cellular signalling pathways. We have previously shown that interaction between the α-cytoplasmic tail of α1β1 integrin and TCPTP activates TCPTP by disrupting an inhibitory intra-molecular bond in TCPTP. Thus, inhibition of the regulatory interaction in TCPTP is a desirable strategy for TCPTP activation and attenuation of oncogenic RTK signalling. However, this is challenging with low molecular weight compounds.

**Methods:**

We developed a high-throughput compatible assay to analyse activity of recombinant TCPTP in vitro. Using this assay we have screened 64280 small molecules to identify novel agonists for TCPTP. Dose-dependent response to TCPTP agonist was performed using the in vitro assay. Inhibition effects and specificity of TCPTP agonists were evaluated using TCPTP expressing and null mouse embryonic fibroblasts. Western blot analysis was used to evaluate attenuation of PDGFRβ and EGFR phosphorylation. Inhibition of VEGF signalling was analysed with VEGF-induced endothelial cell sprouting assays.

**Results:**

From the screen we identified six TCPTP agonists. Two compounds competed with α1-cytoplasmic domain for binding to TCPTP, suggesting that they activate TCPTP similar to α1-cyt by disrupting the intra-molecular bond in TCPTP. Importantly, one of the compounds (spermidine) displayed specificity towards TCPTP in cells, since TCPTP -/- cells were 43-fold more resistant to the compound than TCPTP expressing cells. This compound attenuates PDGFRβ and VEGFR2 signalling in cells in a TCPTP-dependent manner and functions as a negative regulator of EGFR phosphorylation in cancer cells.

**Conclusions:**

In this study we showed that small molecules mimicking TCPTP-α1 interaction can be used as TCPTP agonists. These data provide the first proof-of-concept description of the use of high-throughput screening to identify small molecule PTP activators that could function as RTK antagonists in cells.

## Background

Cellular homeostasis is maintained by the coordinated actions of kinases and phosphatases. Aberrant activation of several kinases due to overexpression, amplification or activating mutations are the underlying causes of many human pathologies like inflammation and cancer [1]. Conversely, loss of the negative regulation exerted by phosphatases may lead to a similar outcome [2]. To date, many kinase inhibitors have been developed and several small molecule inhibitors and function blocking antibodies against receptor tyrosine kinases (RTKs) are already in clinical use to treat different cancers.

TCPTP is a non-receptor protein tyrosine phosphatase (PTP) that is expressed in all tissues throughout development [3]. There are two splice variants of TCPTP that vary in their C-terminal sequence. The longer 48 kDa form (TC48) is localized to the ER whereas the 45 kDa form (TC45) is predominantly present in the nucleus, but it is capable of translocating to the cytoplasm in response to mitogenic stimuli or α1β1-integrin-mediated adhesion to collagen [4-6]. Mice and humans express the 45 kDa form while the 48 kDa form has been identified only in humans [7,8]. TCPTP has been implicated in the negative regulation of several signalling pathways including epidermal growth factor receptor EGFR [4], vascular endothelial growth factor receptor-2 VEGFR2 [9], platelet-derived growth factor receptor beta PDGFRβ [10], signal transducer and activator of transcription-1 STAT1 [11], STAT3 [12], STAT6 [13], the insulin receptor [14], colony stimulating factor 1 signalling [15] and hepatocyte growth factor receptor Met [5]. In addition, two members of the Janus family of tyrosine kinases (JAKs), JAK1 and JAK3, function as TCPTP substrates [16]. TCPTP has recently been established as a negative regulator of SFK, JAK1 and STAT3 signalling during the cell cycle [17].

Recent data has suggested that the production of reactive oxygen species (ROS) is permissive for signalling by RTKs in response to stimuli [18]. Apart from this general mechanism for inhibition of PTPs, in vivo little is known about PTP activation in cells. In the case of Src homology 2-domain containing tyrosine phosphatases 1 and 2 (SHP-1 and SHP-2), binding of the two SH2-domains to phosphotyrosine motifs results in a conformational change and significant activation of the phosphatase [19,20]. In TC45 the positively charged C-terminus was shown to negatively regulate enzyme activity and a truncation mutant (TC37) lacking this region is constitutively active [21]. We have previously shown that TC45 is activated by a collagen-binding integrin α1β1. The positively charged short cytoplasmic tail of α1 integrin (α1-cyt) selectively interacts with the N-terminal part of TC45 and activates it in response to adhesion to collagen via alleviating the autoinhibition by competing with the TC45 C-terminus for binding to the N-terminal half of the protein [6].

Compared to kinases, much less progress has been made in the development of new therapeutics targeting PTPs. Since PTP-1B has an important role in regulating insulin signalling, PTP-1B inhibitors targeting the active site are being developed for treatment of diabetes and obesity [3,22]. Since many PTPs function as negative regulators in cancer [2,23], their agonists could be effective drug targets in oncology. However, this might be challenging since PTP activation may involve disruption of protein-protein interactions which are difficult to break by low molecular weight compounds [24]. In the case of α1-TCPTP interaction a limited number of amino acids in the α1-tail are critical for the interaction [6] making the situation more feasible for targeting with low molecular weight compounds. In the case of p53-MDM2 interaction the development of small-molecule inhibitors has been a success and these compounds induce apoptosis of cancer cells in vivo [25].

Here we have performed a high-throughput screen (HTS) with recombinant TCPTP (TC45) to identify novel activators of the enzyme. We demonstrate that six structurally distinct compounds are capable of activating TC45 in vitro and that one of these, spermidine, functions in cells to inhibit proliferation, PDGFRβ phosphorylation and VEGF-induced angiogenic sprouting in a TC45-dependent manner. We also show that spermidine and mitoxantrone compete with α1-cyt in activating TC45, suggesting that they function by disrupting the inhibitory intra-molecular bond in TCPTP. These lead compounds could be used for rational design of TC45 agonist with improved properties.

## Methods

### Antibodies, siRNAs and reagents

Antibodies against TCPTP (mAb CF4, Calbiochem; 3E2 from M. Tremblay, McGill University, Canada), EGFR phosphotyrosine 1068 (Cell Signaling Technology), PDGFRβ phosphotyrosine 1021 (Santa Cruz), and α-tubulin (Hybridoma Bank) were used. HRP-conjugated second-stage reagents were used, as appropriate. siRNA against TCPTP has previously been shown to be specific to TCPTP and to have identical effects to another TCPTP-targeting oligo [6]. TCPTP siRNA was from Ambion and the All Stars negative control siRNA from Qiagen. Peptides containing the cytoplasmic tails of the α1 or α2 integrins, the integrin-α1 tail fused to the 11-amino acid -long TAT peptide, and scrambled TAT (scrTAT, peptide with the TAT sequence fused to a scramble sequence of the α1-tail amino acids [6]). peptides were synthesized by Innovagen. EGF was purchased from Sigma, and human recombinant PDGF-BB from Cell Signaling Technology. Recombinant TCPTP, its mutants, and the constitutively active TC37 were produced and purified as GST-fusion proteins in Escherichia coli and cleaved from the GST using PreScission according to the manufacturer's instructions (BD Biosciences). DiFMUP (6,8-difluoro-4-methylumbelliferyl phosphate) was purchased from Molecular Probes, and Cell Titer Blue from Promega. Of the small molecule libraries, Spectrum 2000 was from Microsource Discovery Systems, LOPAC (Library of Pharmacologically Active Compounds) 1280 from Sigma, ChemDiv from ChemDiv Inc., ChemBridge from ChemBridge Corporation, and Tripos from Tripos International. Spermidine trihydrochloride, Mitoxantrone, Ruthenium red, and MDL-26,630-trihydrochloride were also from Sigma. Compounds N21 and F12 were no longer available from the library provider and could not be tested further.

### Cells

HeLa cells (ATCC) were maintained in DMEM, 10% FBS, 2 mM L-glutamine, 5% CO2. Primary HUVECs were freshly isolated as described [26] and cultured in EBM2 medium. Only HUVECs passaged ≤ three times were used in this study. They were transfected with siRNA duplexes (100 nM) by nucleofection using Amaxa. Nucleofected HUVECs were harvested for RNA extraction 4 days post-transfection and TCPTP levels were analysed with Taqman qRT-PCR to study transfection efficiency. TCPTP wt (EFM7+/+) and knockout (EFM4-/-) immortalised mouse embryonic fibroblasts (kindly provided by M. Tremblay) were cultured in DMEM, 10% FBS, 2 mM L-glutamine, 5 ug/ml Plasmocin, 5% CO2.

### Western blot assays

Serum-starved HeLa cells or mouse embryonic fibroblasts were left untreated on plastic, stimulated with 10 μM spermidine for 1 h, or in addition treated with 50 ng/ml EGF or PDGF for 5 or 15 min. Cells were lysed in Laemmli's sample buffer and resolved on SDS-PAGE gels for western blot analysis. Western blot bands were quantified using digital image analysis and only non-saturated blots were used. The bands to compare in a blot were enclosed with equal size boxes and pixel intensity was quantified using GeneGenius Bioimaging system and Genetools software (Syngene). Automatic background correction was used.

### Elisa assays

96-well streptavidin plate (Costar) wells were incubated with TBS containing 2.5 μM α1-cytoplasmic tail (biotin-WKIGFFKRPLKKKMEK) or buffer alone at +4°C for 3 h. Non-specific binding was blocked with 2% BSA/TBS-Tween containing 10% FBS overnight at +4°C. Purified recombinant TC45 (cleaved from GST-TC45 as described in [6]) alone or in the presence of indicated concentrations of small molecules, α1-cyt or α2-cyt peptide (α1 cytoplasmic specific sequence lacking the conserved domain RPLKKKMEK or α2 cytoplasmic domain WKLGFFKRKYEMTKNPDEIDETTELSS) was added to the wells and incubated at room temperature for 1 hour. After extensive washing, TC45 protein binding to integrin tail sequences was detected using anti-TCPTP Ab (CF4) and standard horseradish peroxidase -based detection.

### Phosphatase assays

In the in vitro phosphatase assays with purified protein, full-length purified TCPTP (TC45, see above) was incubated in phosphatase reaction buffer (25 mM Hepes, 50 mM NaCl, 1 mM DTT) in the presence or absence of synthetic integrin cytoplasmic tail peptides or the novel small molecule TCPTP activators as indicated. The samples were assayed for phosphatase activity in triplicate using DiFMUP (6,8-difluoro-4-methylumbelliferyl phosphate; Molecular Probes, Eugene, OR) as a substrate. Sample with only reaction buffer and DiFMUP (blank) was used as a control.

### Sprouting assay

Sprouting assays from spheroids were performed as previously described [9], and based on the method described earlier [27]. Briefly, HUVECs were divided into round-bottomed, non-treated 96-wells (Greiner Bio-One), 3000 cells per well, in EBM2 medium with 0.25% methyl cellulose (Sigma). After overnight incubation at +37°C 5 spheroids per treatment were pooled and resuspended in spheroid medium (40% FBS, 0,5% methyl cellulose, plain medium). Collagen gel (20 mM Hepes, 1 × DMEM (Sigma), PureCol collagen (Vitrogen)) was added 1:1 to cell suspension and the mix transferred to 48-wells. After polymerisation, medium +/- VEGF 50 ng/ml, 200 nM ScrTAT or α1TAT-peptide, and 10 uM spermidine were added. Cumulative length of the sprouts around spheroids was quantified using 10× magnification after 24 h incubation.

### High-throughput screening

Five small molecule libraries were screened for TCPTP activators. The library compounds were transferred to 384-well assay plates with a Microlab Star automated liquid handling workstation (Hamilton Robotics) fitted with 100-nl 96-channel pintool (V&P Scientific Inc., San Diego, CA). HTS mode TCPTP dispensation was carried out using a Multidrop Combi dispenser (Thermo Fisher Scientific, Waltham, MA), and the plates were incubated for 10 min. at RT. Background fluorescence was measured during the TCPTP incubation. DiFMUP was added using Multidrop and the plates were incubated for 10 min. at RT. Urea was added to stop the reactions and fluorescence was measured with EnVision 2101 multilabel plate reader (Wallac Oy, PerkinElmer Life Sciences and Analytical Sciences, Turku, Finland). Screening data was analysed and the results normalised using the B-score method, which decreases assay and instrumentation specific variation between datapoints (Brideau et al. 2003) implemented in R, a software environment for statistical computing. (R Development Core Team 2008). B-score hits were compared with background measurement results. Hits in wells having high background signal were discarded as false positive.

### Proliferation assay

TCPTP wt and knockout mouse embryonic fibroblasts were applied on 384-wells, 1500 cells in 35 μl medium (DMEM, 10% FBS, 2 mM L-glutamine) per well. Seven different compound dilutions were prepared in DMSO of Spermidine, Mitoxantrone, Ruthenium Red and MDL. Compounds were added in medium to wells, with 6 replicates of each dilution. The resulting end concentrations of the compounds in the wells were 100 nM, 1 μM, 10 μM, 30 μM, 100 μM, 300 μM and 1 mM. Sample containing only DMSO was used as a negative control. Plates were incubated for 72 h at 37°C, 5% CO_2_. Cell Titer Blue was added to wells and incubated for a further 4 h after which fluorescence at 560 nm was measured with Envision. Data analysis was performed using GraphPad Prism software (GraphPad Software, USA).

### Statistical analyses

Statistical analyses were performed using the two-tailed Student's t-test. All results are expressed as the mean ± SEM. Following P-values were used to show statistical significance: *, P < 0.05; **, P < 0.01; ***, P < 0.001.

## Results

### New TC45 activators found in small molecule screen

**Figure 1:**
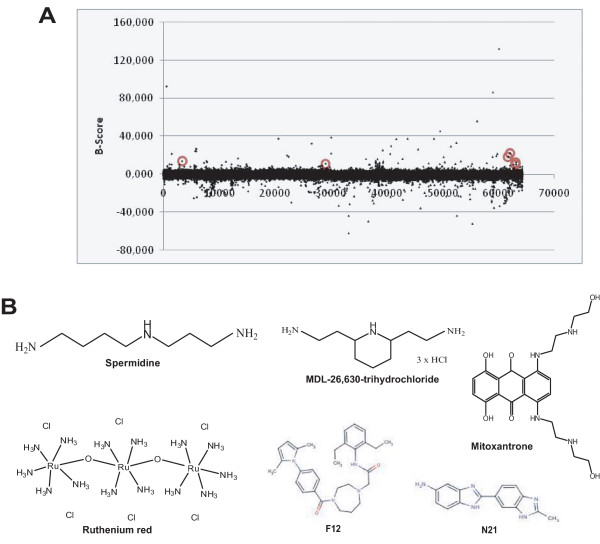
**High-throughput screening**. A. Overview of the high-throughput screening method used. Five small molecule libraries, the Spectrum Collection, LOPAC1280, ChemDiv, ChemBridge and Tripos were screened for TCPTP activators. TCPTP was added to 384-well plates and background fluorescence was measured to control for compound interference due to autofluorescence. DiFMUP phosphatase substrate was added, and the reaction was stopped with urea prior to measuring fluorescence with EnVision. Integrin α1-cyt peptide was added as a positive control to each assay plate. B. Shown are the combined results of all library screens. The data points circled in red represent the 6 confirmed TCPTP activator hits. Data was normalised using the B-score method. C. The chemical structures of the 6 confirmed TCPTP activator hits.

**Figure 2:**
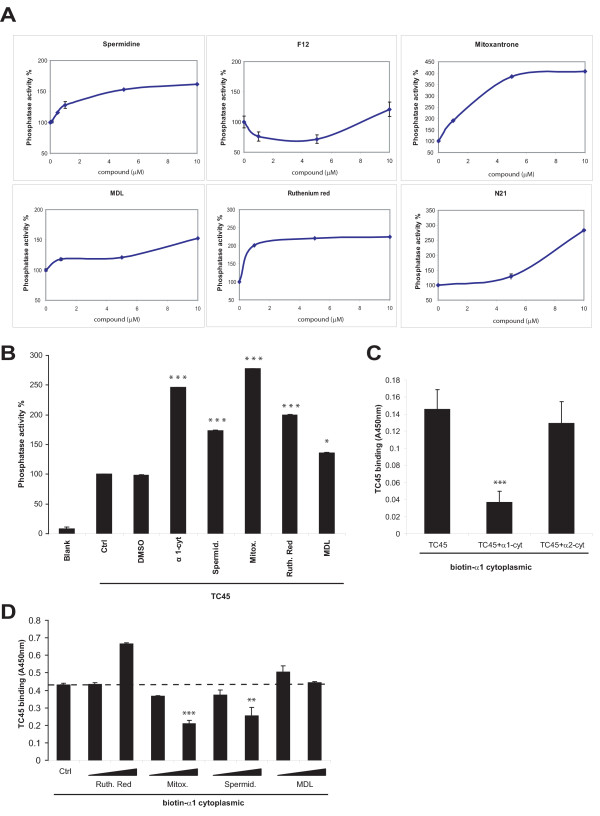
**TCPTP activation and competition assays with α1-cyt with agonists identified in the screen**. A. Small molecules were tested at the indicated concentrations using recombinant TC45 in a DiFMUP phosphatase assay (mean ± SD; n = 3) (due to limited availability N21 was tested only with 10 μM). B. Small molecule activators (10 μM) and α1-cyt peptide (10 μM) were compared for their ability to activate TC45 (mean ± SD; n = 3; ***, p < 0.001). C-D. Direct binding of recombinant purified TC45 to biotinylated integrin α1 cytoplasmic tail was analysed using ELISA assay in the presence or absence of the indicated compounds. C. 11 μM integrin α1 and α2-cytoplasmic domains and D. 1 and 10 μM small molecule compounds were tested for their ability to compete with α1-tail for binding to TC45 (mean ± SD; three parallel measurements from 2 independent experiments **, p < 0.005 ***, p < 0.001).

We performed a high-throughput screen to identify small molecule compounds capable of activating TC45. Five small molecule libraries, Microsource Spectrum with 2000 biologically active and structurally diverse compounds, LOPAC1280 with 1280 pharmaceutically active compounds, Tripos with 6000 compounds, ChemDiv with 25000 compounds and ChemBridge with 30000 compounds were screened in an in vitro phosphatase assay. The assay was based on incubation of purified recombinant TC45 with a fluorescent phosphatase substrate in the presence or absence of small molecule compounds. The compounds were printed on 384-well plates. Compound interference was taken into account by measuring background fluorescence from plates which contained the compounds and the reaction mix, but no TC45. TC45 activity assay was initiated by adding purified phosphatase to the wells and allowing dephosphorylation of phosphatase substrate DiFMUP to proceed for 10 minutes. The dephosphorylation reaction was stopped with urea and the fluorescence measured using a multilabel plate reader (Fig. 1A). Due to the autofluorescence of some compounds, the true hits were revealed by comparing the background and assay fluorescence values (Fig. 1B). After removal of false positives, 213 putative TC45 activators were found in the primary screen (Table 1). Interestingly, many potential inhibitors (data points with a negative B-score) of TC45 were also identified in the primary screen, however these were not followed further in this study. A secondary screen confirmed that six of the compounds activated TC45 in a concentration-dependent manner (Fig. 2A). The molecules capable of activating TC45 in vitro were spermidine trihydrochloride (spermidine), mitoxantrone, ruthenium red, MDL-26,630-trihydrochloride (indicated hereafter as MDL), N21 (C_15_H_13_N_5_) and F12 (C_30_H_38_N_4_O_2_), the chemical structures of which are shown in Fig. 1C. Out of these mitoxantrone was most potent showing on average a 3.3-fold activation of TC45 which was comparable with the 2.4-fold activation by α1-cyt, a known activator of TC45 [6]. (Fig. 2B).

**Table 1:** Hit rate of TCPTP activators after removal of false positives due to compound interference.

Library	Compounds	Primary hits	Confirmed activators
Tripos	6000	5	1
ChemDiv	25000	23	1
ChemBridge	30000	167	0
Microsource Spectrum	2000	16	2
Sigma Lopac	1280	2	2

Total	64280	213	6

Primary hits were individually picked from the libraries and screened again in HTS. Reproducing hits were confirmed with different concentrations resulting in total of six validated TCPTP activators.

### Spermidine and mitoxantrone activate TC45 via a mechanism similar to a1-cyt

More detailed information on the molecular mechanism governing activation of TC45 is needed to facilitate rational drug design of activating molecules. Thus, we investigated the mechanism behind the small molecule -mediated TC45 activation. Based on our previous results, integrin α1 cytoplasmic tail residues 1164-1179 interact with the amino-terminal part of TC45 activating it by alleviating the proposed autoregulatory interaction between the C- and N-terminus of the protein [6]. Since α1 cytoplasmic tail is positively charged (RPLKKKMEK) and also the majority of the identified small molecular activators of TC45 carry positively charged amine-groups, we investigated whether the compounds would compete with α1-cyt for binding of TC45. Purified recombinant TC45 interacts directly with biotinylated α1-cyt peptide and the interaction is sensitive to competition with 11 μM α1-peptide but not α2-peptide (Fig. 2C). Interestingly, spermidine and mitoxantrone competed with the solid-phase bound α1-cyt in a concentration-dependent manner (Fig. 2D), suggesting that these compounds unlike MDL and Ruthenium Red activate TCPTP by binding to the same site as the integrin cytoplasmic tail.

### Spermidine inhibits serum-induced cell proliferation in a TC45-dependent manner

**Figure 3:**
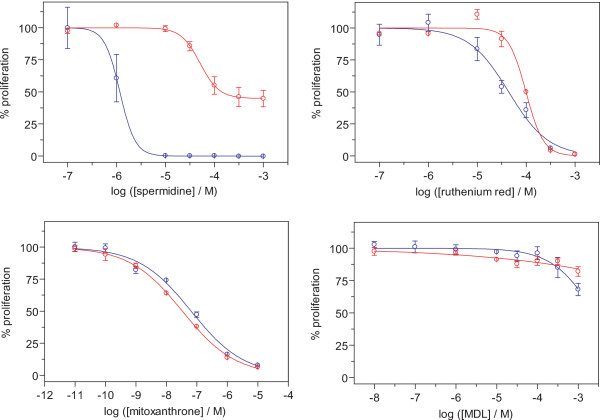
**Dose-dependent responses of TCPTP expressing and null MEFs to TCPTP agonist**. Four TC45 activating compounds: spermidine, ruthenium red, mitoxanthrone; and MDL-26,630-trihydrocloride were tested for their effect on cell proliferation in TCPTP wild-type (O) and knockout (O) mouse embryonic fibroblasts (MEFs). Wildtype and knockout MEFs were incubated in serum-containing medium in the presence of the HTS-identified compounds, as indicated for 72 h. Cells were detected with Cell Titer Blue reagent, and the non-linear regression analysis was done using GraphPad Prism (mean ± SD of 3 experiments with similar results).

The majority of TCPTP activators identified here are previously described molecules with known cellular targets other than TC45. Therefore, it was important to characterize whether they influence cell behaviour in a TC45-dependent manner. TCPTP is a known negative regulator of many mitogenic signalling pathways and acute silencing of TCPTP induces cell proliferation in cancer cells and alterations in signalling pathways [9,10,28,29]. To study the TC45-dependency of these compounds, we tested four of the compounds for their effect on cell proliferation in TC45 knockout and wt mouse embryonic fibroblasts. The cells were incubated with the compounds at indicated concentrations for 72 h and live cells were detected. Strikingly, spermidine displayed specificity towards TC45, since TC45 knockout cells were 43-fold more resistant to the compound than wt cells, suggesting that the presence of TCPTP makes the wt cells more sensitive to the drug regarding proliferation (Fig. 3). Since spermidine can be oxidized in serum-containing culture [30], we cannot rule out the possibility that the spermidine effect is in fact a function of a spermidine-derived compound. The other three compounds did not show specificity towards TCPTP and either inhibited cell proliferation equally well in both cell types (ruthenium red and mitoxantrone) or had no significant effect on proliferation (MDL) at the investigated concentrations.

### Spermidine regulates EGFR and PDGFRβ signalling via TC45

**Figure 4:**
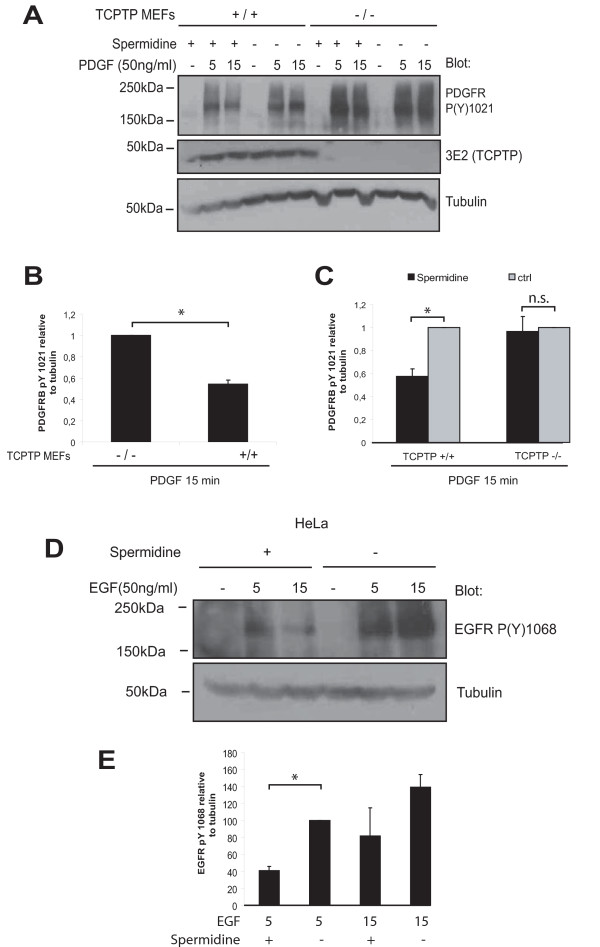
**Inhibitory effects of spermidine on RTK signalling**. A. PDGF-induced PDGFRβ phosphorylation is inhibited by spermidine in a TCPTP-dependent manner. Serum-starved TCPTP wt (+/+) and knockout (-/-) MEFs were incubated for 1 h in the presence or absence of spermidine and stimulated with PDGF as indicated. Cell lysates were resolved on SDS-PAGE and probed for phosphorylation at PDGFRβ pY1021. TCPTP (3E2) and tubulin blots serve as controls for TCPTP expression in wt and KO cells, and loading, respectively. B, C. Quantitation of PDGFRβ pY1021 phosphorylation levels normalized against tubulin in TC45 wt and KO cells (mean ± SEM; n = 2; *, P ≤ 0.05) B. or control- and 10 μM spermidine-treated cells (mean ± SEM; n = 3; *, P ≤ 0.05) C. following 15 minute PDGF treatment are shown. D. Spermidine attenuates EGF-induced EGFR signalling. Serum-starved HeLa cells were treated with 10 μM spermidine for 1 h, or left untreated, and stimulated with or without 50 ng/ml EGF for 5 or 15 minutes. Cell lysates were resolved on SDS-PAGE and immunoblotted with phospho-specific EGFR antibody. Tubulin was used as the loading control. A representative experiment out of three with similar results is shown. E. Quantitation of EGFR pY1068 phosphorylation levels normalized against tubulin in 10 μM spermidine treated and control EGF-stimulated HeLa cells (mean ± SEM; n = 3; *, P ≤ 0.05).

TC45 negatively and site-selectively regulates PDGFRβ phosphorylation [10]. To investigate whether spermidine could attenuate PDGFRβ signalling in a TC45-dependent manner, we used TC45 knockout and wt mouse embryonic fibroblasts. We studied the phosphorylation levels of PDGFRβ Tyr 1021 in spermidine-treated cells in the presence or absence of PDGF-BB, after overnight starvation. In line with previous data [10], we observed that PDGF-BB induced 45 ± 4% higher phosphorylation of PDGFRβ in TC45 knockout cells compared to wt cells (Fig. 4A, B). Importantly, spermidine was able to attenuate PDGF-induced PDGFRβ phosphorylation by 42 ± 6% in TC45 wt cells whereas no inhibition was detected in TC45 negative cells (Fig. 4A, C). Therefore, activation of TC45 with spermidine results in TC45-dependent negative regulation of a well-characterized TC45 target in cells.

Previously we have shown that collagen-induced activation of TC45 by integrin α1β1 attenuates EGFR phosphorylation at multiple sites [6]. Since our results with spermidine implied that it can function as a TC45 activator in mouse cells, we tested if spermidine would have an effect on EGFR phosphorylation in human cancer (HeLa) cells. Treatment of the cells with spermidine was sufficient to significantly attenuate EGFR phosphorylation in cells (Fig. 4D, E). The observed attenuation was similar to the one achieved with TC45 activation by cell-membrane permeable TAT-α1-cyt peptide in this cell line [6]. Taken together, these data indicate that spermidine-induced activation of TC45 can be used to inhibit signalling of TC45 target RTKs like PDGFRβ and EGFR in human and mouse cells.

### Endothelial cell sprouting is inhibited by spermidine

**Figure 5:**
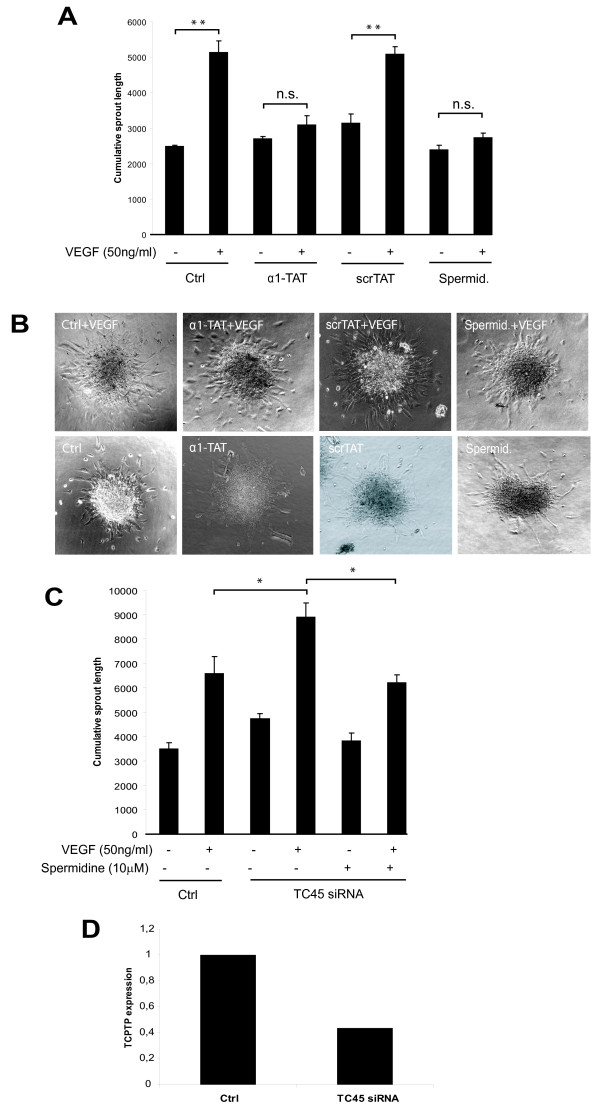
**Inhibition effects of spermidine on VEGF-induced endothelial cell sprouting**. A. HUVEC spheroids embedded in three-dimensional collagen gel were treated with VEGF (50 ng/ml), 200 nM TAT peptides and 10 μM spermidine as indicated. Spheroids were analyzed after 24 h with 10× magnification and the cumulative sprout length was quantified using image analysis. Shown is a representative of three similar experiments (mean ± SEM; n = 4-5 spheroids per treatment; **, p ≤ 0.01). B. Representative images show HUVEC spheroids after 24 h incubation with the indicated treatments taken with 10× magnification. C. HUVEC spheroid assay performed as in A, but with TCPTP siRNA -treated HUVEC (mean ± SEM; n = 4-5 spheroids per treatment; *, p ≤ 0.05). D. qRT-PCR quantitation of the TCPTP knockdown in HUVECs used in C.

Recently we showed that VEGFR2 is under the negative regulation of TC45 and α1-integrin in endothelial cells (Mattila et al. 2008). We demonstrated that activation of TC45 with cell-membrane permeable α1-TAT peptide significantly reduced the length of the VEGF-induced sprouts in three-dimensional cultures of HUVECs in a TC45-dependent manner [9]. Here we tested the effect of spermidine on VEGF-induced sprout formation in HUVEC spheroids (Korff, Augustin 1999). Also in this model spermidine and α1-TAT but not scrTAT (a control peptide with TAT-sequence fused to a scramble sequence containing the α1 amino acids in random order) were able to significantly inhibit VEGF-induced sprouting in HUVECs (Fig. 5A, B). The basal sprouting detected in the absence of VEGF was not affected by spermidine (Fig. 5A, B). Importantly, the ability of spermidine to inhibit VEGF-induced endothelial sprouting was at least partially dependent on TC45. We used a previously characterized TC45-specific siRNA that shows identical effects to another independent siRNA oligo and does not influence levels of other PTPs like SHP-2 [6,9]. Silencing of TC45 (Fig. 5D) increased VEGF-induced sprouting in TC45-silenced cells compared to control cells by 35 ± 7% (Fig. 5C). Furthermore, in TC45-silenced cells spermidine inhibited VEGF-induced sprouting by 43 ± 5% compared to the 89 ± 3% inhibition by spermidine in control cells. The somewhat limited effects induced by the TCPTP siRNA in these experiments are most likely due to moderate TCPTP silencing achieved in HUVEC (Fig. 5D). These results demonstrate that in human primary endothelial cells VEGF-induced sprouting is inhibited by spermidine and that TC45 expression is required for efficient spermidine-mediated inhibition of VEGF signalling.

## Discussion

At present, massive efforts are being undertaken to generate drugs (tyrosine kinase inhibitors, TKIs) that inhibit RTKs playing a central role in human diseases like cancer. Many of the targeted specific therapies have failed and a growing view is that compounds hitting several targets at the same time may be more beneficial than single specificity agents (Fojo 2008). Furthermore, many patients develop resistance to the clinically used inhibitors, which could be circumvented by targeting the same pathway via other means. TCPTP is a well-characterized negative regulator of several cancer relevant RTKs including EGFR [4], cMet [5], PDGFRβ [10], Insulin receptor [14], and VEGFR2 [9]. In addition, TCPTP is an important player in immune cells, suggesting that activating it might be advantageous also in treating inflammatory conditions [23,28]. Here we have applied a large scale HT-screen to identify small molecules capable of activating TCPTP. Our results demonstrate that one of the compounds, spermidine, is capable of activating TC45 in vitro. Activation of TC45 with spermidine attenuates EGFR, PDGFRβ and VEGFR2 signalling in different cell types and models. This suggests that TCPTP activators could possess the desired capacity of targeting multiple pathways and circumventing known acquired resistance mechanisms to TKIs like mutations in the ATP binding sites of RTKs.

The chemical structures of the small molecule activators of TC45 are presented in Fig. 1C. They are all discrete entities and the main obvious common feature is that most of the compounds contain positively charged amine-groups. Mitoxantrone is a well-known agent used in chemotherapy. It interferes with both DNA and RNA and is a potent inhibitor of topoisomerase II [31]. Mitoxantrone is used in treatment of prostate cancer and acute nonlymphocytic leukemia (ANLL), as well as multiple sclerosis [32-34]. Therefore, it was not surprising that its cytotoxic effects were not TC45-dependent in cells (Fig. 3). However, it was the most potent activator of TC45 in vitro and this could be due to its long, rigid and flat structure, which is sufficiently large to achieve high-affinity binding to TCPTP at a site that overlaps the α1-cyt binding site. Thus structural information from this hit could be useful if TCPTP activators are to be developed further.

Spermidine is a long, flexible, positively charged polyamine involved in cellular metabolism [35]. The fact that spermidine inhibits cell proliferation and attenuates RTK signalling in a TC45-dependent manner is somewhat surprising, considering that all cells contain substantial amounts of at least one of the polyamines, putrescine, spermidine, and spermine [36]. However, based on our data it is clear that acute treatment with micromolar extracellular spermidine, which is taken up by an effective transport system and possibly metabolised to spermidine derivatives, is sufficient to result in activation of TC45 and subsequent negative regulation of signalling pathways. This would be in line with a previous report which demonstrated that spermidine or spermidine related compounds, which have been derived from spermidine via oxidation, are cytotoxic to cells with upregulated growth pathways [30] and previous reports demonstrating spermidine effects on RTK signaling [37,38]. At present, the direct interaction between TCPTP and α1-cytoplasmic tail is the only mechanism of TCPTP activation in cells that has been characterized in detail [6]. The structure of spermidine resembles that of α1-cyt in that both contain positively charged amine groups, and this may be reflected in its ability to trigger cellular responses similar to α1-TAT peptide (Fig. 5). This is further supported by our finding that spermidine interacts with TC45 at overlapping sites. It is obvious that none of the molecules identified here are suitable therapeutic agents for the modulation of TCPTP function in human malignancies. This is mainly due to two reasons. First, the identified compounds have also other targets in cells. Second, they may present limited membrane permeability due to a highly ionized nature and the highly flexible, non-drug-like structures of some of the compounds. However, they provide important structural information and may function as starting points for the development of TCPTP activators in the future.

## Conclusion

To the best of our knowledge similar PTP agonist screens have not been published and thus the hits add to the few examples of PTP activating molecules described thus far [39,40]. In conclusion, the small molecule TCPTP activators described here improve our understanding of the molecular mechanism of α1-cyt-mediated activation of TC45 and can allow rational design of improved TC45 activators that could become clinically relevant therapeutics in the future.

## Competing interests

The authors declare that they have no competing interests.

## Authors' contributions

EM carried out the phosphatase assays, western blots, sprouting assays and also drafted the manuscript. HM and NS carried out the HTS and the secondary screening. PK carried out the data normalization and bioinformatics. PH helped with the proliferation assays, ST carried out the ELISA assays, MP co-ordinated and designed the HTS. JI designed the study, analysed the results and revised the manuscript. All authors read and approved the final manuscript.

## Pre-publication history

The pre-publication history for this paper can be accessed here:

http://www.biomedcentral.com/1471-2407/10/7/prepub

## References

[B1] Hunter T Signaling--2000 and beyond Cell 2000 100 1 113 127 10.1016/S0092-8674(00)81688-8 10647936

[B2] Ostman A Hellberg C Bohmer FD Protein-tyrosine phosphatases and cancer Nat Rev Cancer 2006 6 4 307 320 10.1038/nrc1837 16557282

[B3] Alonso A Sasin J Bottini N Friedberg I Osterman A Godzik A Hunter T Dixon J Mustelin T Protein tyrosine phosphatases in the human genome Cell 2004 117 6 699 711 10.1016/j.cell.2004.05.018 15186772

[B4] Tiganis T Bennett AM Ravichandran KS Tonks NK Epidermal growth factor receptor and the adaptor protein p52Shc are specific substrates of T-cell protein tyrosine phosphatase Mol Cell Biol 1998 18 3 1622 1634 9488479 10.1128/mcb.18.3.1622 PMC108877

[B5] Sangwan V Paliouras GN Abella JV Dube N Monast A Tremblay ML Park M Regulation of the Met receptor-tyrosine kinase by the protein-tyrosine phosphatase 1B and T-cell phosphatase J Biol Chem 2008 283 49 34374 34383 10.1074/jbc.M805916200 18819921 PMC2662243

[B6] Mattila E Pellinen T Nevo J Vuoriluoto K Arjonen A Ivaska J Negative regulation of EGFR signalling through integrin-alpha1beta1-mediated activation of protein tyrosine phosphatase TCPTP Nat Cell Biol 2005 7 1 78 85 10.1038/ncb1209 15592458

[B7] Mosinger B Jr Tillmann U Westphal H Tremblay ML Cloning and characterization of a mouse cDNA encoding a cytoplasmic protein-tyrosine-phosphatase Proc Natl Acad Sci USA 1992 89 2 499 503 10.1073/pnas.89.2.499 1731319 PMC48266

[B8] Cool DE Tonks NK Charbonneau H Walsh KA Fischer EH Krebs EG cDNA isolated from a human T-cell library encodes a member of the protein-tyrosine-phosphatase family Proc Natl Acad Sci USA 1989 86 14 5257 5261 10.1073/pnas.86.14.5257 2546150 PMC297600

[B9] Mattila E Auvinen K Salmi M Ivaska J The protein tyrosine phosphatase TCPTP controls VEGFR2 signalling J Cell Sci 2008 121 Pt 21 3570 3580 10.1242/jcs.031898 18840653

[B10] Persson C Savenhed C Bourdeau A Tremblay ML Markova B Bohmer FD Haj FG Neel BG Elson A Heldin CH Ronnstrand L Ostman A Hellberg C Site-selective regulation of platelet-derived growth factor beta receptor tyrosine phosphorylation by T-cell protein tyrosine phosphatase Mol Cell Biol 2004 24 5 2190 2201 10.1128/MCB.24.5.2190-2201.2004 14966296 PMC350555

[B11] ten Hoeve J de Jesus Ibarra-Sanchez M Fu Y Zhu W Tremblay M David M Shuai K Identification of a nuclear Stat1 protein tyrosine phosphatase Mol Cell Biol 2002 22 16 5662 5668 10.1128/MCB.22.16.5662-5668.2002 12138178 PMC133976

[B12] Yamamoto T Sekine Y Kashima K Kubota A Sato N Aoki N Matsuda T The nuclear isoform of protein-tyrosine phosphatase TC-PTP regulates interleukin-6-mediated signaling pathway through STAT3 dephosphorylation Biochem Biophys Res Commun 2002 297 4 811 817 10.1016/S0006-291X(02)02291-X 12359225

[B13] Lu X Chen J Sasmono RT Hsi ED Sarosiek KA Tiganis T Lossos IS T-cell protein tyrosine phosphatase, distinctively expressed in activated-B-cell-like diffuse large B-cell lymphomas, is the nuclear phosphatase of STAT6 Mol Cell Biol 2007 27 6 2166 2179 10.1128/MCB.01234-06 17210636 PMC1820499

[B14] Galic S Klingler-Hoffmann M Fodero-Tavoletti MT Puryer MA Meng TC Tonks NK Tiganis T Regulation of insulin receptor signaling by the protein tyrosine phosphatase TCPTP Mol Cell Biol 2003 23 6 2096 2108 10.1128/MCB.23.6.2096-2108.2003 12612081 PMC149470

[B15] Simoncic PD Bourdeau A Lee-Loy A Rohrschneider LR Tremblay ML Stanley ER McGlade CJ T-cell protein tyrosine phosphatase (Tcptp) is a negative regulator of colony-stimulating factor 1 signaling and macrophage differentiation Mol Cell Biol 2006 26 11 4149 4160 10.1128/MCB.01932-05 16705167 PMC1489091

[B16] Simoncic PD Lee-Loy A Barber DL Tremblay ML McGlade CJ The T cell protein tyrosine phosphatase is a negative regulator of janus family kinases 1 and 3 Curr Biol 2002 12 6 446 453 10.1016/S0960-9822(02)00697-8 11909529

[B17] Shields BJ Court NW Hauser C Bukczynska PE Tiganis T Cell cycle-dependent regulation of SFK, JAK1 and STAT3 signalling by the protein tyrosine phosphatase TCPTP Cell Cycle 2008 7 21 3405 3416 18948751 10.4161/cc.7.21.6950

[B18] Finkel T Oxidant signals and oxidative stress Curr Opin Cell Biol 2003 15 2 247 254 10.1016/S0955-0674(03)00002-4 12648682

[B19] Paez JG Janne PA Lee JC Tracy S Greulich H Gabriel S Herman P Kaye FJ Lindeman N Boggon TJ Naoki K Sasaki H Fujii Y Eck MJ Sellers WR Johnson BE Meyerson M EGFR mutations in lung cancer: correlation with clinical response to gefitinib therapy Science 2004 304 5676 1497 1500 10.1126/science.1099314 15118125

[B20] Pei D Lorenz U Klingmuller U Neel BG Walsh CT Intramolecular regulation of protein tyrosine phosphatase SH-PTP1: a new function for Src homology 2 domains Biochemistry 1994 33 51 15483 15493 10.1021/bi00255a030 7528537

[B21] Hao L Tiganis T Tonks NK Charbonneau H The noncatalytic C-terminal segment of the T cell protein tyrosine phosphatase regulates activity via an intramolecular mechanism J Biol Chem 1997 272 46 29322 29329 10.1074/jbc.272.46.29322 9361013

[B22] Zhang S Zhang ZY PTP1B as a drug target: recent developments in PTP1B inhibitor discovery Drug Discov Today 2007 12 9-10 373 381 10.1016/j.drudis.2007.03.011 17467573

[B23] Tonks NK Protein tyrosine phosphatases: from genes, to function, to disease Nat Rev Mol Cell Biol 2006 7 11 833 846 10.1038/nrm2039 17057753

[B24] Arkin MR Wells JA Small-molecule inhibitors of protein-protein interactions: progressing towards the dream Nat Rev Drug Discov 2004 3 4 301 317 10.1038/nrd1343 15060526

[B25] Vassilev LT Vu BT Graves B Carvajal D Podlaski F Filipovic Z Kong N Kammlott U Lukacs C Klein C Fotouhi N Liu EA In vivo activation of the p53 pathway by small-molecule antagonists of MDM2 Science 2004 303 5659 844 848 10.1126/science.1092472 14704432

[B26] Koskinen K Vainio PJ Smith DJ Pihlavisto M Yla-Herttuala S Jalkanen S Salmi M Granulocyte transmigration through the endothelium is regulated by the oxidase activity of vascular adhesion protein-1 (VAP-1) Blood 2004 103 9 3388 3395 10.1182/blood-2003-09-3275 14726375

[B27] Korff T Augustin HG Tensional forces in fibrillar extracellular matrices control directional capillary sprouting J Cell Sci 1999 112 Pt 19 3249 3258 10504330 10.1242/jcs.112.19.3249

[B28] van Vliet C Bukczynska PE Puryer MA Sadek CM Shields BJ Tremblay ML Tiganis T Selective regulation of tumor necrosis factor-induced Erk signaling by Src family kinases and the T cell protein tyrosine phosphatase Nat Immunol 2005 6 3 253 260 10.1038/ni1169 15696169

[B29] Tiganis T Kemp BE Tonks NK The protein-tyrosine phosphatase TCPTP regulates epidermal growth factor receptor-mediated and phosphatidylinositol 3-kinase-dependent signaling J Biol Chem 1999 274 39 27768 27775 10.1074/jbc.274.39.27768 10488121

[B30] Otsuka H The toxic effect of spermidine on normal and transformed cells J Cell Sci 1971 9 1 71 84 4327649 10.1242/jcs.9.1.71

[B31] Fox EJ Mechanism of action of mitoxantrone Neurology 2004 63 12 Suppl 6 S15 18 15623664 10.1212/wnl.63.12_suppl_6.s15

[B32] Comi G Induction vs. escalating therapy in multiple sclerosis: practical implications Neurol Sci 2008 29 Suppl 2 S253 255 10.1007/s10072-008-0954-x 18690509

[B33] Tallman MS Gilliland DG Rowe JM Drug therapy for acute myeloid leukemia Blood 2005 106 4 1154 1163 10.1182/blood-2005-01-0178 15870183

[B34] Dreicer R Current status of cytotoxic chemotherapy in patients with metastatic prostate cancer Urol Oncol 2008 26 4 426 429 18593622 10.1016/j.urolonc.2007.11.005

[B35] Moinard C Cynober L de Bandt JP Polyamines: metabolism and implications in human diseases Clin Nutr 2005 24 2 184 197 10.1016/j.clnu.2004.11.001 15784477

[B36] Heby O Role of polyamines in the control of cell proliferation and differentiation Differentiation 1981 19 1 1 20 10.1111/j.1432-0436.1981.tb01123.x 6173280

[B37] Faaland CA Laskin JD Thomas TJ Inhibition of epidermal growth factor-stimulated EGF receptor tyrosine kinase activity in A431 human epidermoid carcinoma cells by polyamines Cell Growth Differ 1995 6 2 115 121 7756170

[B38] Fujita-Yamaguchi Y Sacks DB McDonald JM Sahal D Kathuria S Effect of basic polycations and proteins on purified insulin receptor. Insulin-independent activation of the receptor tyrosine-specific protein kinase by poly(L-lysine) Biochem J 1989 263 3 813 822 2557012 10.1042/bj2630813 PMC1133503

[B39] Takahashi T Takahashi K Mernaugh RL Tsuboi N Liu H Daniel TO A monoclonal antibody against CD148, a receptor-like tyrosine phosphatase, inhibits endothelial-cell growth and angiogenesis Blood 2006 108 4 1234 1242 10.1182/blood-2005-10-4296 16597593 PMC1895872

[B40] Tomic S Greiser U Lammers R Kharitonenkov A Imyanitov E Ullrich A Bohmer FD Association of SH2 domain protein tyrosine phosphatases with the epidermal growth factor receptor in human tumor cells. Phosphatidic acid activates receptor dephosphorylation by PTP1C J Biol Chem 1995 270 36 21277 21284 10.1074/jbc.270.36.21277 7673163

